# Supplementary material for: Are carbon emissions trading and green financial instruments synergistic? -Comprehensive quantitative research based on content analysis
Source: PLoS One. 2024 Mar 7;19(3):e0298601. doi: 10.1371/journal.pone.0298601 (PMC10919601; doi:10.1371/journal.pone.0298601)
Supplement: S1 Data — (ZIP) [file pone.0298601.s001.zip › Data/地级市Z_F工作报告.zip!/╔╜╢1⁄2/╝├─■╩╨/└┤╫╘╣1⁄2╓┌║┼í╛┬φ┐╦╩2╛▌═°í┐_2017.docx]

各位代表：

现在，我代表济宁市人民政府向大会报告工作，请予审议，并请市政协委员提出意见。

一、过去五年工作回顾

五年来，在中共济宁市委坚强领导下，我们坚决贯彻党中央、国务院和省委、省政府及市委决策部署，抓住用好系列重大机遇，大力实施重大产业、城市建设、基础设施和民生实事项目，倾力办好事关全局的大事要事，各项工作取得新成绩，市十六届人大历次会议确定的目标任务顺利完成。

（一）综合实力实现新跨越。地区生产总值连跨两个千亿台阶，达到4302亿元，是2011年的1.5倍；地方财政收入达到391.5亿元，基本实现翻番；固定资产投资累计突破万亿大关、达到12706亿元；城乡居民收入年均增长9.5%、11.4%。县域经济倍增计划全面实现，所有县市区地方财政收入实现翻番。

（二）经济结构不断优化。争取省政府出台加快推进济宁资源型城市可持续发展政策，从7个方面助推我市转型升级，新型产业体系加快形成。三次产业结构调整为11.2：45.3：43.5。粮食生产连年稳定在百亿斤以上。新增规模以上工业企业1213家、达到2624家，增量居全省首位。服务业税收占比达到49.1%，增加值占比提高8.6个百分点，年均增幅居全省第2位。高新技术产业产值占比达到29.9%。2个产业规模超千亿、5个超500亿。2家企业进入中国跨国公司100强，4家进入中国企业500强。信息产业高端起步，惠普、甲骨文、中兴、华为等一批知名IT企业集群落户，被确定为省信息技术产业基地。

（三）改革开放成效明显。大力推进“放管服”改革，实施行政审批流程再造，削减行政审批事项329项、压减55.4%。深化商事制度改革，新增市场主体27万户。市县政府机构改革、公务用车改革、不动产登记改革、农村信用社银行化改革圆满完成。土地承包经营权确权登记颁证、集体林权制度改革基本完成。对接“一带一路”战略，实现进出口总额265亿美元，境外投资70亿美元、居全省第2位，如意集团、太阳纸业等一批境外投资项目进展顺利。

（四）城乡面貌显著变化。成功实施行政区划调整，中心城区形成“一城五区”发展格局，进入国家2类大城市行列。常住人口城镇化率达到55.3%、提高11.2个百分点，增幅连续4年居全省第1位。实施“精致济宁”系列工程，改造老旧小区180个、11.1万户，建成“四小工程”19个，开工地下综合管廊15.4公里，引水入城项目竣工。泗河综合开发全域启动，济徐高速建成通车，滨湖大道全线贯通，新增公路通车里程3116公里，完成农村“户户通”2.2万公里，8成以上行政村通公交。建成美丽乡村示范片区31个，90%以上的村达到环境整洁村标准，城乡环卫一体化经验全国推广，荣获“中国人居环境范例奖”。成功创建全国绿化模范城市，全国双拥模范城市实现七连冠，全国文明城市创建获得提名，国家卫生城市创建顺利通过国家级技术评估。

（五）文化旅游成果丰硕。孔子学院总部体验基地、干部政德教育基地和济宁干部政德教育学院、孟子研究院批准设立。传统文化六进普及、道德提升等七大工程深入实施，儒学进乡村活动蓬勃开展，被授予山东“四德工程”建设示范市。县乡村文化设施实现全覆盖。尼山圣境、市文化中心等一批重点项目进展顺利，新增省级以上文化产业示范园9家，规模以上文化企业达到407家、居全省第2位，新建亿元以上旅游项目66个，完成投资414.6亿元，旅游消费总额突破600亿元。

（六）生态环境持续改善。国家生态保护与建设示范区创建扎实推进。完善“治用保”水污染防治体系，119个治污项目全部完成，南四湖跻身全国14个水质良好湖泊行列。持续开展大气污染防治集中攻坚，空气质量综合改善幅度居全省前列。新增造林110万亩，森林覆盖率达到30.8%，建成国家级生态乡镇43个。关停淘汰落后产能企业371家，万元GDP能耗下降17%。

（七）社会事业全面进步。民生支出1811亿元、年均增长13.5%。精准扶贫成效明显，17.1万人实现脱贫。新增城镇就业38万人，农村劳动力转移就业34万人，扶持创业6.5万人，荣获首批全国创业先进城市。五项社会保险制度实现全覆盖，连年提高企业退休人员养老金标准，城乡低保分别提高78%、146%。实施“大班额”和“全面改薄”系列工程，新改扩建学校1254所，12个县市区全部通过义务教育均衡发展国家认定。先看病后付费、分级诊疗模式和“四位一体”养老服务经验全国推广。成功举办第23届省运会和第9届省残运会。平安济宁建设成效明显，“天网”工程基本建成，被授予“全国社会管理综合治理优秀市”，群众满意度连年提升。国防动员、人民防空、民兵预备役、国家安全、广播影视、新闻出版、民族宗教、外事侨务、公积金、粮食、物价、地震、气象、水文、档案、史志、老龄、残疾人、红十字、慈善和妇女儿童等各项工作取得新成绩。

五年来，我们扎实开展党的群众路线教育实践活动、“三严三实”专题教育、“两学一做”学习教育，驰而不息纠“四风”，政府自身建设不断加强。全面推进依法行政，荣获第三届“中国法治政府奖”。主动接受人大法律监督、政协民主监督，办理议案建议725件、政协提案2427件。完善重大行政决策程序，强化政务公开，权力运行更加规范透明。严格落实党风廉政建设“两个责任”和“一岗双责”，加强行政监察、审计监督，全市上下形成了风清气正、干事创业的良好氛围。

各位代表，刚刚过去的2016年极不平凡。一年来，我们围绕稳定经济增势，成立经济运行调度指挥中心，“一业一策”制定帮扶措施，经济运行稳中向好，地区生产总值、地方财政收入增幅高于全省0.4、0.5个百分点，工业利税、利润同比增长7%、7.7%。我们围绕推进供给侧结构性改革，圆满完成“三去一降一补”年度任务，去除粗钢产能30万吨，核减煤炭产能1558万吨；完成商品房去库存1133万平方米，去化周期降至合理区间；实体经济新增贷款273.9亿元，处置银行不良贷款居全省前列；综合为企业减负80亿元左右。我们围绕培育发展新动能，实施创新驱动战略，如意集团获批国家纺纱工程技术研究中心、工业设计中心，荣获第四届中国工业大奖、中国优秀工业设计金奖。联诚集团中标国家工业强基工程，泰丰液压、山推股份入围全国首批制造业单项冠军示范（培育）企业，惠普软件产业园、省激光研究所被认定为国家国际科技合作基地。国家级科研平台达到11家，引进“千人计划”专家33人。我们围绕夯实城市规划建设基础，制定了21项导则，城市规划建设管理标准体系进一步完善。城市总体规划提前半年获省政府批复，都市区和主城区快速交通体系规划编制完成，轨道交通线网规划通过省技术审查，海绵城市、地下综合管廊专项规划完成修编，城区控规实现全覆盖。《济宁市泗河保护管理条例》颁布实施，为进一步保护开发利用好泗河提供了坚实保障。我们围绕突破民生重点工作，在全省率先出台《济宁市大气污染防治条例》，率先实现乡镇街道、化工园区空气质量智能化监测站全覆盖，大气治理网格化监管体系全面建立，空气质量持续改善，“蓝天白云、繁星闪烁”天数同比增加65天，居全省首位。解决教育大班额完成投资46亿元，相当于过去4年中小学校投资总和，竣工面积153.2万平方米，相当于过去3年建设总和。完成棚改5.56万户、危改1.02万户、厕改31.5万户，均超额完成省定任务。我们围绕抓住放大系列机遇，积极做好对上争取工作，曲阜优秀传统文化传承发展示范区进入国家“十三五”规划，国土资源部核减我市采煤塌陷基本农田17.12万亩，国家能源局批复我市建设100万千瓦光伏发电示范基地，跨湖高速、董梁高速等一批重大基础设施项目开工建设。

这些成绩的取得，是省委、省政府和市委正确领导的结果，得益于历届领导班子奠定的坚实基础，离不开市人大、市政协的监督支持，凝聚着广大干部群众的心血汗水。在此，谨代表市人民政府，向历届老领导、老同志，向全市各族人民、各界人士、驻济部队指战员、武警官兵、公安干警和人大代表、政协委员致以崇高敬意，向关心支持济宁发展的港澳台同胞、海外侨胞和国际友人表示衷心感谢！

各位代表，我们也清醒地看到，全市经济社会发展仍存在不少问题。主要是，经济结构性矛盾突出，产业层次不高，新旧动能转换不快，“三去一降一补”任务艰巨；中心城市辐射带动能力不强，城镇化水平偏低，县域经济发展不平衡；银行不良贷款率偏高，各种金融风险不容忽视；煤炭消耗量大，大气污染防治任重道远；民生领域存在不少短板，社会治理还有薄弱环节；一些政府工作人员法治观念淡薄，公共服务效率不高，隐形“四风”问题仍不同程度存在。对这些问题，我们一定高度重视，精准施策，加快解决。

二、新一届政府主要任务

今后五年是全面建成小康社会的决胜阶段。推进发展既面临宏观环境复杂多变、经济运行压力加大的严峻形势，也面临新一轮产业变革蓄势待发、新增长动能孕育形成的战略机遇。市十三次党代会确定了我市今后五年的宏伟蓝图，凝聚起科学发展、转型发展共识。只要我们齐心协力、埋头苦干，就一定能够开创济宁各项工作新局面。

主要任务目标是：地区生产总值突破6000亿元、地方财政收入突破600亿元，人均地区生产总值突破70000元，城乡居民收入分别达到45000元、22000元，城镇化率达到65%以上，全面建成小康社会，实现全面转型振兴。

——建设淮海经济区中心城市。都市区深度融合、同城发展，中心城区扩容提质、首位度持续提升，各板块发展由“圈层扩散型”向“集聚紧凑型”转变，“水陆铁空”综合交通体系更加完备，区域产业创新中心、文化教育中心、生活服务中心地位基本确立，城市聚集承载和辐射带动能力显著增强，基本建成充满活力和竞争力的淮海经济区中心城市。

——建设鲁西科学发展排头城市。鲁西经济、文化、金融、商贸物流、科技教育和先进制造业“六大中心”基本建成，经济实力、创新能力和综合竞争力居于龙头地位，成为山东西部最具实力、最富活力的科学发展高地，在西部经济隆起带加快崛起中发挥引领带动作用。

——建设转型发展先行城市。以创新驱动引领全面转型，产业结构实现“三二一”重大转变，科技进步贡献率达到60%以上，高新技术产业、战略性新兴产业产值占比达到35%、40%左右，主导产业形成万亿级规模，信息产业、文化旅游产业成为引领转型发展的支柱产业。国家生态保护与建设示范区取得丰硕成果，森林覆盖率达到33%以上，空气质量优良天数持续增加，国家森林城市创建成功。

——建设弘扬优秀传统文化示范城市。主动担当弘扬中华优秀传统文化历史重任，挖掘和用好丰富的儒家文化资源，文化软实力和影响力持续提升。文化强市首善之区建设迈出坚实步伐，曲阜文化建设示范区取得重大进展，优秀传统文化传承发展体系基本形成，覆盖城乡的公共文化服务体系更加完善。

——建设宜居宜业幸福城市。公共财力重点用于保障改善民生。城市建成区绿地率达到40%以上，棚改、老旧小区改造和中心城区雨污分流基本完成。行政村公交通行率达到90%以上，镇镇通天然气全面实现，农村集中连片供水基本实现全覆盖，农村贫困人口全部脱贫。城乡一体化进程加快推进，公共服务均等化水平显著提升，群众获得感进一步增强。

实现上述目标，必须坚决贯彻落实中央和省委、省政府及市委决策部署，按照市十三次党代会确定的指导思想、目标任务，紧扣济宁发展的阶段性特征，自觉践行五大发展理念，在发展格局调整中强化特色、厚植优势，在区域合作竞争中扬长补短、转型发展。必须全面深化供给侧结构性改革，聚力转型振兴，聚焦补齐短板，从制约我市发展的重要领域和关键环节着手，既补硬短板也补软短板，既补发展短板也补制度短板，打造转型发展新优势。必须坚持以人民为中心的发展思想，集中力量做好普惠性、基础性、兜底性民生建设，在解决群众最关心、最现实的利益问题中促进公平共享，在富裕百姓、造福百姓中增进民生福祉。必须不断提高发展的境界标准，坚持站高一步、想深一步、看远一步，打破按部就班、四平八稳、惯性发展的旧观念，树立敢于负责、勇于担当、善于突破的新风气，形成定了就干、真抓实干、埋头苦干的浓厚氛围。

我们坚信，通过全市850万人民的艰苦奋斗、不懈努力，历史悠久、文化璀璨的孔孟之乡必将谱写出新的精彩篇章，一个产业强、环境美、百姓富的新济宁必将崛起在齐鲁大地！

三、2017年政府工作总体安排

今年是新一届政府开局之年，我们要全面贯彻党的十八大和十八届三中、四中、五中、六中全会精神，深入学习贯彻习近平总书记系列重要讲话和视察山东视察济宁重要讲话、重要指示精神，统筹推进“五位一体”总体布局，协调推进“四个全面”战略布局，坚持稳中求进工作总基调，以新发展理念为引领，以供给侧结构性改革为主线，大力实施创新驱动、工业强市、文旅突破、开放带动、城乡一体、生态立市六大战略，全面做好稳增长、促改革、调结构、惠民生、防风险各项工作，促进全市经济平稳健康发展和社会和谐稳定。主要预期目标为：地区生产总值增长8%左右，地方财政收入增长8.5%左右，固定资产投资增长11.5%左右，社会消费品零售总额增长10.5%左右，城镇和农村居民人均可支配收入分别增长8%和8.5%左右，城镇登记失业率控制在4%以内，物价上涨总水平控制在3%左右，节能减排完成省定目标。重点做好四个方面的工作。

（一）经济发展的重点工作。牢固树立工业要集中、产业要集聚、土地要集约发展理念，坚持一手抓规模与转型，一手抓创新与技改，推动实体经济加快向绿色、高端和智能方向发展。坚持把招商引资作为经济工作的生命线，着力在体制机制、政策考核上实现新突破，围绕龙头企业以商招商，拉长产业链条，形成产业集聚，做大产业集群；坚持把项目建设作为经济工作的出发点和落脚点，搞好项目引进、项目推进和后续服务，让好的项目享受好的资源，促进项目快落地、快建设、快达效；坚持把环境营造作为经济工作的强力支撑，优化法治、人文、政策、生态环境，完善负面清单、权力清单、责任清单，打造法治化、国际化、便利化营商环境，使济宁成为海内外客商向往的投资兴业热土。

一是提高农业供给质量。加快发展“新六产”，推进农村一二三产深度融合，抓好泗水、金乡、嘉祥省级试点。实施藏粮于地、藏粮于技战略，新建高产高效示范方26.2万亩。扩大“粮改饲”试点范围，扶持特色农业规模化发展。提升济宁、邹城国家农业科技园区示范带动效应，新建市级现代农业示范园20处。发挥泗河全域开发对观光农业的牵引作用，打造滨河美丽乡村示范带。扶持圣丰等一批种业公司发展壮大。推进农业标准化生产，认证“三品一标”产品50个，新增大田托管面积30万亩。完善农产品从田间到餐桌的安全监管体系，保障群众“舌尖上的安全”。启动新一轮农田水利、环南四湖生态渔塘改造等重点工程，完成农田水利项目3641个。

二是推动工业转型升级。坚持工业强市不动摇，深入实施“中国制造2025济宁行动计划”，高起点编制“9+6”重点产业发展规划。推动以信息产业为重点的新兴产业跨越发展，依托惠普、甲骨文、中兴、华为等IT产业巨头，对接世界行业龙头，大力发展信息制造业。实施“6501”信息产业培植计划，加快四大信息产业园建设，筹建大数据产业园、大数据交易所。培育“互联网＋”产业发展新业态，加速“两化”融合，提升高端装备、生物医药、节能环保、新能源、新材料等新兴产业智能化水平，建设一批产业集群、骨干企业和示范基地。提质发展优势传统产业，启动新一轮高水平技术改造，市级层面集中实施100个重大技改项目，全市技改投资突破1000亿元。依托龙头企业，采取兼并重组、合资合作等措施，重点建设工程机械、纺织服装、化工橡胶、高端造纸、食品加工等主导产业补链项目，带动传统产业向价值链高端跃升。推进煤炭利用清洁化，支持兖矿等煤炭企业建设清洁型煤生产基地。依托鲁抗、辰欣、方健等医药企业，高标准建设济宁高新区、兖州区、邹城市医疗健康产业园。突出抓好要素保障，精准对接国家、省专项资金，设立新兴产业和煤炭等传统产业转型发展基金。严格落实国家降税减费措施，确保企业负担只减不增。严格执行安全、环保、能耗等法律法规，坚定不移化解过剩产能。

三是发展现代服务业。继续实施服务业三年行动计划，加快推动生产性服务业向专业化和价值链高端延伸，生活性服务业向精细化和高品质转变，支持每个县市区打造1处现代服务业集聚区，服务业增加值占比提高1个百分点以上。文化旅游业，实施文化产业“十大业态”提升工程和“文化+”计划，促进文化与科技、旅游、金融、创意深度融合，打造3个重点文化产业园区，培育4家大型企业，孵化一批中小微企业。以创建国家级旅游业改革创新先行区为引领，实施“全景济宁、全域旅游”战略，组建文化旅游集团，搞好环微山湖生态湿地公园规划建设，推进微山湖创建5A级景区，加快鱼台孟楼湿地建设，争创无障碍文化旅游城市。金融业，加大招商力度，完善组织体系，再引进3家以上金融机构，支持济宁银行设立跨区域分行。推进政银企对接常态化，加大金融对实体经济支持力度。妥善化解担保圈风险，加快不良贷款处置，严厉打击恶意逃废金融债务行为，规范发展互联网金融。现代物流业，发挥国家级物流节点城市功能，以运河“黄金水道”、主要港口和济宁新机场、兖州货运站、高铁站为依托，优化交通枢纽与物流空间布局，加快推进临港、临空、临铁物流园区建设，培育壮大20家市级现代物流企业，打造区域物流集散中心，尽快形成公路运输、疏港运输、铁路专列、航空货运“四位一体”物流新格局。电子商务业，深化与阿里巴巴、京东、苏宁等知名电商合作，推动商贸、供销、邮政、电商互联互通，开展电子商务示范项目创建，建设电商基地20个，扶持每个县培育2个以上电商产业园、30家以上电商企业，电商交易额突破1400亿元。养老服务业，积极应对人口老龄化，推动公办养老机构改革，引导社会资本进入养老服务业，加快西部滨河养老产业示范园规划建设，构建居家为基础、社区为依托、机构为补充、医养相结合的养老服务体系，努力让全市老人颐养天年。

四是加大科技创新力度。持续加大科研院所引进力度，加强与大院大所的深度合作，构建以产学研为主线、“产业+企业+平台+人才+金融”的区域创新体系。突出企业主体地位，引导企业加大研发投入，转化100项科技成果，形成重点科技企业创新梯队。推进重大创新平台建设，新建院士工作站5个，新增工程技术研究中心50家、企业技术中心及工业设计中心38家。启动实施“沃土”计划，规划建设国家级人力资源服务产业园，建好省级人才改革试验区，引进一批高层次、高技能中青年人才，形成“引进人才—培育企业—催生产业”链式效应。创新人才评价、甄别、奖励和收入分配、生活保障机制，确保人才引得来、留得住。构建“亲”“清”新型政商关系，真心实意为企业家服务，有计划组织百名企业负责人赴境外培训、千名企业高管到高校研修。

五是实施质量强市战略。以创建“全国质量强市示范城市”为引领，推进“好品济宁”区域品牌建设，新增驰著名商标17个、国家地理标志证明商标5个、省级以上名牌产品8个。加大对知名品牌、驰著名商标的扶持力度，建好“济宁品牌产品主题馆”，打造面向海外的线上公共服务平台。企业、政府、社会各方面一起努力，让玉堂酱园、微山湖、金蜂糕点、广育堂等老字号焕发新生机，如意、金太阳、华勤、菱花、山推、荣信、鲁抗等大品牌漂洋过海，金乡大蒜、鱼台大米、泗水粉条、微山麻鸭等地理标志农产品走进千家万户，辰欣、晨阳、卡松、圣阳等众多成长型品牌叫得更响，打造一批孔孟之乡的金招牌、新代言。

六是深化改革扩大开放。推进农业农村改革，细化落实农村土地“三权分置”办法，抓好农村集体产权制度改革。完善国有企业公司治理结构，积极发展混合所有制经济。深化财税体制改革，坚持公共财政原则，逐步实现基本公共服务均等化“五个基本统一”。推进企业规范化改制，完成300家以上改制任务，15家以上企业挂牌上市。统筹抓好投融资、事业单位、城市管理等重点领域改革。构筑双向开放新优势，完善扩大出口政策，加强重点行业动态跟踪，推动出口产品结构和贸易主体结构“双优化”，提高“三自三高”产品出口比重。加快济宁综合保税区、保税物流中心申建步伐，发挥济宁（金乡）内陆港作用，为外贸企业提供便利化通关服务。深化与世界500强企业对接，鼓励企业融入“一带一路”等国家战略，拓宽国际合作领域。

七是加快县域经济发展。实施民营经济“百企护航”行动，筛选20家主营业务收入过10亿元的骨干企业，围绕“防风险、稳运行、促裂变”搞好服务；筛选30家主营业务收入过5亿元的科技型企业，支持其依靠“创新驱动、项目带动、品牌拉动”突破发展瓶颈；筛选50家主营业务收入过亿元的成长型企业，着眼“扩融资、减税费、研新品”强化帮扶措施，通过分类扶持，助推重点企业发展，提升民营经济质量规模。支持县市区发挥比较优势，实施一批建链、强链、补链项目，做强主导产业，做大龙头企业，带动一批配套型中小企业加速成长。加大对梁山专用汽车、微山新能源、汶上服装、嘉祥手套等产业扶持力度，每个县培育1-2个产业集群，形成多点支撑、多业并举的县域经济板块。坚持“外引”与“内孵”并举，支持产业基础相对薄弱的县市区，把主攻方向放在招商引资上，推进全产业链、专业化、精细化招商，弥补产业发展短板；鼓励产业基础较好的县市区，在抓好招商引资的同时，集聚要素资源，扶持本土企业发展壮大。出台经济开发区提升发展五年行动计划，编制新一轮园区发展规划，建立开放创新、区镇分开、产城融合的园区发展模式和绩效薪酬体系。加快搭建科技创新平台、检验检测共享平台，完善公共服务配套，提升承载能力。支持兖州工业园区、邹城工业园区争创国家级开发区，争取济宁（金乡）新材料产业园跻身省级园区，加快鱼台张黄循环经济示范园区建设，推进济宁高新区向国家级开发区第一方阵迈进。

（二）城乡建设的主要任务。按照“精明增长、紧凑城市”发展理念，加快精致济宁建设，推进以人为核心的新型城镇化进程，实现15万农业转移人口和13万城中村、城边村居民市民化。

一是统筹城乡规划建设。优化“一城五区”生产力布局，支持各板块发挥优势、相向融合，构筑都市区、中心城区、中等城市、特色乡镇、新型农村社区五级新型城镇体系。顺应城市发展规律，强化空间管控，严禁盲目扩规模铺摊子，推动城市发展向内涵提升转变。抓好重点区段、河道水系、交通体系、地下空间和采煤塌陷地等规划建设，打造沿河、沿湖、沿历史街巷的生态人文环境。推进老城区、新城区和各功能区联动发展，完善太白湖新区公共服务配套，启动石桥特色城区建设；推动任城老城区有机更新，实施西部滨河新区整体开发；加快兖州西部城区建设，与济北新区、高新区全面对接；高标准建设经开区中央商务区、商贸物流园区，壮大特色产业园规模；支持高新区建设城市复合中心，推进产城融合，打造“圣地智谷”。

二是增强城市服务功能。着眼解决“城市病”，推进城市“双修”，完善便民服务功能，构建“15分钟社区服务圈”和“5分钟便民生活圈”。重点抓好“五大提升工程”。实施道路提升工程，改造琵琶山路等21条道路，打通红星西路等24条断头路，开工火炬路日菏铁路跨线桥等3座大型桥梁；新增公共停车位1万个，建设充电站24座、充电桩1300个，更换新能源公交车100辆，增加公共自行车5000辆，提升城市疏堵保畅能力。实施棚改提升工程，完成6.59万户棚改、2.7万户老旧小区改造任务，消化商品房库存800万平方米，保持房地产市场“量增价稳”。实施管网提升工程，建设20个海绵城市示范项目，建成地下综合管廊10公里，完成雨污分流47公里，改造燃气、供热管网30公里。实施绿化提升工程，加大环城林带等十大绿化项目推进力度，加快凤凰台公园、儿童公园和高新区百里绿道、经开区十字绿廊建设步伐，提升城市主次干道绿化水平，结合棚改完成20个小绿地、小游园建设任务。丰富城市植物结构，常绿植物不低于50%，新增造林20万亩、绿化面积200公顷、湿地保护修复10万亩、绿色通道300公里。实施水系提升工程，改造洸府河、大运河等8条河流生态景观，完成泗河综合开发堤防工程，抓好采煤塌陷地生态修复，与平原水库、湿地公园有机结合，打造“环城生态绿带”和慢行系统，增添城市休闲新空间，逐步形成“一环八水绕济宁，十二明珠映古城”特色水城风貌。

三是构建综合交通体系。跨湖高速、董梁高速完成部分路基桥涵工程，鲁南高铁、济宁新机场开工建设。抓好城市主干道与机场、高铁、高速公路的对接，提速建设西二环、北二环，完成轨道交通规划报批，启动建设机场高速，尽快形成“田字形”高速路网结构，全市新增公路里程200公里，加快构建中心城区与周边城市、重要交通节点的快速路网体系，提高城际通达能力。

四是提升城市建管水平。开展“工程质量管理年”活动，从严落实七方主体责任，全面提高工程建设质量。集中治理违法建设，完成60%拆违任务。落实物业管理条例，推进垃圾分类处理设施建设，垃圾无害化处置率达到95%以上。实施城市家具提升工程，完善公交站牌、导引标识、便民坐凳等公共设施。深化智慧城管建设，实现数字化城管市域全覆盖，提高城市管理规范化、精细化、智能化水平，打造精致、优美、整洁、有序的城市环境。

五是加快小城镇和美丽乡村建设。实施特色小镇培育工程，市县联动集中打造3个特色小镇，支持每个县打造1个重点镇。深化镇驻地环境综合整治，启动镇镇通天然气工程，开展农村分片集中供水试点，让农民群众喝上放心水。完成40万户农村改厕任务，提升31个美丽乡村示范片区、100个示范村建设水平，统筹推进绿化、亮化、美化等重点任务，充分彰显田园风光、乡村风情。

（三）社会发展的优先事项。牢固树立公共财政观念，严控“三公”经费等一般性支出，集中财力解决人民群众最关心、最现实的问题。

打赢脱贫攻坚战。深化精准扶贫“六大攻坚行动”，加大医疗、教育、产业救助力度，强化政府兜底保障，实现脱贫3.4万人，确保三年任务两年完成。严格脱贫攻坚考核评估，坚决防止弄虚作假，切实做到“真扶贫”、“扶真贫”。

实施就业优先战略。落实促进高校毕业生就业创业政策，统筹推进大学生“三支一扶”等基层服务项目，采取财税、金融、培训等措施，强化对困难人员的就业援助。构建“基地+创投”创业模式，积极培育众创、众包、众扶、众筹，年内培训1万人，扶持创业1.3万人，带动就业6.5万人。开展农村“双创”活动，加强对农村致富带头人、新型农民的培训，培育职业农民2000人。

完善社会保障制度。进一步提高城乡居民基础养老金、城乡低保、农村五保和基本医疗保险标准，健全社会救助与物价上涨挂钩联动机制，不断提高困难群众生活水平。实施全民参保登记计划，完善职工大病保险制度，开展长期护理保险试点。做好留守儿童、孤寡老人救助工作，新建城市社区日间照料中心30个、村级互助养老院100个、新增养老服务床位5000张。

促进教育均衡发展。深入推进“大班额”和“全面改薄”工程，优化中小学和幼儿园布局，加强优质高中建设，新改扩建城镇中小学105所，新增面积166.3万平方米、学位11.4万个、教师5900人，有效破解“大班额”、“择校热”等问题。实施“名师名校长”工程，加大教师交流力度。加强“招院引校”工作，支持曲阜师范大学、济宁医学院、济宁学院等高等院校加快发展，加力推动职业教育增规模上水平，优化学科结构，建设特色专业，打造教育名城。

发展卫生健康事业。把全民健康放在优先发展战略地位，大力实施“名医、名科、名院”工程，促进城乡医疗卫生资源均衡布局，市中医院新院投入使用，市第一人民医院高新区分院二期工程、太白湖市立医院开工建设，新增床位4000张，80%以上的乡镇卫生院和社区卫生服务中心达到标准化水平。实施中医惠民工程，依托市中医院老院区筹建针灸推拿医院，全市所有乡镇卫生院和社区卫生服务中心全部建成“国医堂”综合服务区，用尽可能少的费用维护居民健康。开展出生人口性别比综合整治，抓好妇幼健康服务机构标准化建设，增加产科、儿科医护人员和床位设施。推动全民健身与全民健康深度融合。加强食品药品安全监管，建设“食安济宁”。

提升生态环境质量。树立“绿水青山就是金山银山”理念，多管齐下增蓝天、保碧水、护净土。科学布局生产、生活、生态三大空间，严守生态、城市开发边界和耕地保护三条红线。健全大气污染联防联控机制，深化压煤、抑尘、控车、禁烧、增绿治理措施，完成78台燃煤锅炉超低排放、5家焦化企业干熄焦改造任务，开展加油站清理整顿，落实国六标准汽柴油供应，强化高排放车辆监管，加快能源和产业结构调整，PM2.5、PM10、二氧化硫、二氧化氮分别改善10%、11%、10%、3%以上。全面推行“河长制”，整治城乡黑臭水体，对涉水污染源实行排放总量与浓度“双控制”，抓好环南四湖水污染综合治理，完成19个重点治污项目建设，保障重要水源地、河道水系及南水北调水质安全。严格落实“土十条”，开展重污染土壤治理和修复试点，做好第二次污染源普查，提高项目准入门槛，杜绝新上高耗能、高排放项目，坚决关停有毒有害高污染企业。

扎实推进文化建设。高标准建设曲阜优秀传统文化传承发展示范区，聘请一流团队搞好控规和专项规划编制，策划实施国学小镇、旅游特色小镇等一批牵动性项目。加强文物保护，有序推进文化古迹和历史街区保护建设。抓好尼山圣境、三孔彩绘等项目建设，孔子博物馆建成开放。持续提升孔子学院总部体验基地功能，加快干部政德教育学院、孟子研究院建设步伐，办好国际孔子文化节、世界儒学大会、国际中学生儒学辩论大会、论语大会，扩大济宁文化知名度和影响力。广泛开展文明创建活动，争创全国文明城市。加大公共文化基础设施投入，提升100处文化广场、100处文化大院，基层综合性文化服务中心达标率超过80%。实施精品创作生产工程，抓好第二轮修志工作。推动全民阅读，让济宁成为书香四溢的文化之城。

着力创新社会治理。推广“文化善治”模式，以传统文化温润人心，夯实社会治理基础。抓好村（居）民委员会换届工作，增强社区自治和服务功能。深化平安济宁建设，加快“和为贵”调解室和专职调解员队伍建设。加强和改进信访工作，常态化开展领导干部公开接访。深入开展“大快严”专项治理，确保安全生产形势持续稳定。强化监测预警和风险防控，完善突发事件联动处置机制。继续做好双拥共建工作，推进军民融合发展。提升民族宗教、外事侨务等各项工作水平。

（四）政府建设的主要举措。增强“四个意识”特别是核心意识、看齐意识，始终在思想上政治上行动上同以习近平同志为核心的党中央保持高度一致，确保政令畅通、令行禁止。

努力克服本领恐慌。开展学习型机关建设活动，政府机关单位带头建设学习型组织。深入学习习近平总书记治国理政新理念新思想新战略，学习经济、城建、科技、金融等各方面知识，增强适应互联网时代和经济新常态的能力水平。强化干部精准培训，开展城乡规划、创新驱动、产业升级等专题培训，提高政府工作人员专业素养。鼓励干部拜群众为师、向群众学习，定期开展走访活动，了解社情民意，增强政府工作的针对性、实效性。

提升政务服务效能。优化行政审批流程，探索实施多图、多评联审和“一图评审”制度，试行“一会三函”模式，审批时限再提速50%以上。强化事中事后监管，实现“双随机一公开”制度全覆盖。完善政务服务中心职能，打造便民服务综合平台，真正让群众“进一个门、办所有事”。推行“互联网+政务服务”，借力大数据构建“云服务政府”。加强社会征信体系建设，全面公布市县乡公共服务清单，探索开展政务公开标准化规范化试点。发挥好“12345”市长公开电话桥梁作用，搭建政府与群众更好更畅通的沟通平台。

严格依法履行职责。坚持法治思维和依法行政，自觉接受人大及其常委会的法律监督和人民政协的民主监督，认真办理议案建议和政协提案。严格落实重大行政决策法定程序，主动听取人大代表、政协委员和法律顾问的意见建议。坚决制止一切形象工程、政绩工程，反对任何统计数据造假。落实执法责任制和责任追究制，纠正不当行政行为，促进社会公平正义。

大力弘扬实干作风。对“三重工作”实行项目制管理，推行“清单制+责任制”工作方法，明确主体责任、时间表、路线图，倒排工期、挂图作战。强化督导问责，做到有部署必有督导，有失职必严问责。坚持约束与激励并举，健全容错纠错机制，旗帜鲜明地为担当者担当，为干事者撑腰。坚持党管干部、事业用人原则，打破论资排辈观念，切实做到优者上、庸者下、劣者汰。下决心精简会议文件，控制会议规模，压缩会议时间，为基层减压松绑，让基层干部有更多精力谋发展、抓落实。

强化党风廉政建设。严格落实全面从严治党主体责任，坚持“一岗双责”，健全廉政风险防控机制，以减权限权、创新监管等举措减少寻租空间，铲除滋生腐败土壤。抓住公共权力运行、公共资源配置、公共资金分配等重点领域，推进审计监督全覆盖。加强政府投资类项目监管，严格招投标程序，强化预算约束，从严控制工程造价，严查侵害群众利益的不正之风和腐败问题，坚持不懈地把党风廉政建设和反腐败工作推向深入，切实做到干部清正、政府清廉。

各位代表，蓝图已绘就，扬帆正当时。让我们更加紧密地团结在以习近平同志为核心的党中央周围，在中共济宁市委的坚强领导下，齐心协力，攻坚克难，撸起袖子加油干，为全面建成小康社会、实现全面转型振兴、谱写济宁改革发展新篇章而努力奋斗！
